# Rethinking preventive medicine education after COVID-19: bridging training gaps in public health practice

**DOI:** 10.3389/fmed.2026.1852914

**Published:** 2026-07-30

**Authors:** Yu-long Cao, Jiao Shan, Xiao-yuan Bao, Meng Jin, Wei Huai, Yi-cheng Jin, Yi-xi Jin, Zhi-ning Zhang, Ji-qiu Kuang

**Affiliations:** 1 Department of Hospital-Acquired Infection Control, Peking University People's Hospital, Beijing, China; 2 Department of Hospital-Acquired Infection Control, Beijing Jishuitan Hospital, Capital Medical University, Beijing, China; 3 Medical Informatics Center, Institute of Advanced Clinical Medicine, Peking University, Beijing, China; 4 Department of Emergency, Peking University Third Hospital, Beijing, China; 5 School of General Studies, Columbia University, New York, NY, United States; 6 Khoury College of Computer Science, Northeastern University, Seattle, WA, United States; 7 Graduate School of Medicine, Kyoto University, Kyoto, Japan

**Keywords:** capacity building, medicine education, preventive medicine, public health, training

## Abstract

The COVID-19 pandemic exposed persistent structural weaknesses in public health systems worldwide, particularly in relation to workforce preparedness and the translation of training into practice. These shortcomings have renewed attention to the role of preventive medicine education, which is increasingly expected to respond to complex and rapidly evolving health challenges. Drawing on literature published since the COVID-19 pandemic and recent reforms in China, particularly within the framework of the “Healthy China 2030” initiative, and considering broader international developments, this mini-review examines how preventive medicine training is being reshaped. Recent shifts include a greater emphasis on competency-oriented approaches, expanded practice-based learning, and the integration of interdisciplinary perspectives. However, graduates of preventive medicine programs frequently perceived gaps between academic training and practical public health competencies, particularly in field epidemiology and emergency response preparedness. Building on these observations, we outline a forward-looking perspective that highlights the potential role of systems thinking, adaptive training models, and emerging forms of agentic learning. While these approaches appear promising, their practical implementation and long-term impact have yet to be fully established. Overall, this review seeks to contribute to ongoing discussions on how preventive medicine education can better support the development of a responsive and resilient public health workforce in the post-pandemic context.

## 1 Introduction

Public health systems worldwide were placed under considerable strain during the COVID-19 pandemic, bringing into sharper focus longstanding weaknesses in workforce capacity, emergency preparedness, and the integration of clinical and population-based approaches to health. While these challenges are not entirely new, the scale and persistence of the pandemic appear to have exposed limitations that had previously received less sustained attention.

Preventive medicine, positioned at the intersection of epidemiology, health promotion, and disease prevention, is often regarded as central to strengthening public health systems. In practice, however, its educational foundations have not always kept pace with evolving demands. Traditional training models continue to place substantial emphasis on theoretical instruction, often at the expense of applied competencies and real-world problem-solving capacity (1). This imbalance remains a recurring concern in discussions of public health workforce development.

In China, the “Healthy China 2030” initiative has provided a policy framework for accelerating reforms in preventive medicine education, with a stated shift toward prevention-oriented health systems. Comparable transitions can also be observed in other settings, where competency-oriented training, interdisciplinary approaches, and experiential learning models are increasingly emphasized (2). Even so, the extent to which these reforms have translated into meaningful improvements in practice remains uncertain.

Existing studies, including those examined in this review, point to several persistent challenges, such as fragmented curricular structures, limited integration of practice-based training, and gaps in the development of core public health competencies. Against this backdrop, this review seeks to examine recent developments in preventive medicine education while also reflecting on areas that remain insufficiently addressed (3). Drawing on recent reforms in China, particularly within the Healthy China 2030 framework, while also considering educational developments reported in North America, Europe, and other international settings, this review explores both shared and context-specific trends in preventive medicine education reform.

## 2 Literature identification and synthesis strategy

A targeted literature search was conducted to identify recent studies relevant to preventive medicine education and public health workforce development. Publications were retrieved primarily from PubMed and Web of Science databases between January 2020 and March 2026 using combinations of keywords including “preventive medicine education,” “public health education,” “competency-based education,” “public health workforce,” “practice-based learning,” “field training,” “artificial intelligence,” and “medical education reform.”

Priority was given to peer-reviewed articles, policy analyses, systematic reviews, and educational evaluation studies published in English. Articles were screened for relevance to post-pandemic educational transformation, workforce competency development, curriculum reform, experiential learning, and emerging digital technologies in public health training. The selected literature was thematically analyzed and organized into four major domains: current educational challenges, emerging reform trends, practice-based training approaches, and the evolving role of artificial intelligence (AI) in preventive medicine education. To mitigate potential selection bias, priority was given to peer-reviewed studies, policy analyses, systematic reviews, and educational evaluation studies that directly address preventive medicine education reform or post-pandemic public health workforce development. Heterogeneous sources were included to capture a range of perspectives, including empirical findings, conceptual frameworks, and policy-driven initiatives.

## 3 Current challenges in preventive medicine education

Recent literature emphasizes the importance of systems thinking and adaptive capacity among public health students. Reflecting on these insights, we recommend that training programs incorporate targeted modules to cultivate these competencies in preparation for evolving public health challenges. A particularly persistent issue lies in the imbalance between theoretical instruction and practical training. Although this imbalance has long been acknowledged, it remains only partially addressed in many programs. Didactic, lecture-based approaches are still widely used, often resulting in graduates who are less well prepared for field-based epidemiological work, emergency response, and context-dependent decision-making (4).

A further concern relates to the organization of curricula, which in many cases remain divided along traditional disciplinary lines, including epidemiology, environmental health, and biostatistics. While such structures provide foundational knowledge, they may not sufficiently support the development of integrated thinking. As a result, students may find it challenging to apply systems-oriented perspectives when confronted with complex public health issues such as pandemics, climate change, and non-communicable diseases (5). This limitation is particularly evident in settings where cross-disciplinary collaboration is required but not adequately reflected in training.

While many of these challenges are observed internationally, their manifestations differ across contexts. In China, discussions frequently emphasize the imbalance between preventive and clinical medicine training, as well as insufficient field-based public health exposure. In contrast, educational reforms in North America and Europe have increasingly focused on competency assessment, interprofessional education, and workforce adaptability. Despite these differences, a common concern across regions remains the need to better align educational outcomes with real-world public health practice.

In addition, the availability and quality of practice-based training opportunities vary considerably across institutions. In some cases, partnerships with public health agencies are limited or inconsistently implemented, which can restrict students' exposure to real-world public health scenarios. This gap may, in turn, affect their ability to translate theoretical knowledge into actionable interventions, especially in emergency contexts where timely decision-making is critical. Faculty-related factors also warrant attention. Although many educators have strong academic training, opportunities for sustained engagement in frontline public health practice may be limited. This can create challenges in delivering practice-oriented instruction and mentoring students in applied settings. Taken together, these issues suggest that current educational models, while evolving, may not yet fully align with the competencies required for effective public health practice (6).

## 4 Emerging trends in educational reform

In response to the limitations outlined above, a range of educational reforms has been introduced across different settings, although their scope and implementation vary considerably. Among these, competency-based education (CBE) has attracted sustained attention as a framework that shifts the focus from knowledge acquisition to the development of measurable and practice-relevant skills. Commonly emphasized competencies include epidemiological analysis, risk communication, leadership, and emergency response (7). While this approach provides a clearer structure for training outcomes, questions remain regarding how consistently such competencies are defined and assessed in practice.

At the same time, there has been growing interest in practice-oriented learning models, including problem-based learning, case-based learning, and simulation-based training (8). These approaches are often intended to move beyond passive knowledge transfer by engaging students in scenario-based problem-solving. In principle, they offer opportunities to strengthen critical thinking and decision-making under conditions of uncertainty. However, their effectiveness appears to vary according to local implementation conditions. For example, Tarfa et al. (9) found that outcomes of public health workforce training programs were strongly influenced by faculty engagement, institutional support, and implementation fidelity.

Interdisciplinary integration has also been increasingly emphasized in recent curriculum reforms. Preventive medicine training programs are beginning to incorporate elements from fields such as data science, behavioral science, environmental health, and health policy. This reflects a broader recognition that contemporary public health challenges are shaped by multiple interacting determinants rather than single risk factors. Even so, integrating these domains into a coherent training framework remains a complex task, and the extent to which such integration translates into improved competencies is not yet fully clear (10).

In parallel, digital technologies are playing an expanding role in reshaping public health education. Online platforms, virtual simulations, and data-driven tools have created more flexible and scalable learning environments, particularly in contexts where traditional field training is limited. More recently, AI and machine learning have begun to be incorporated into areas such as epidemiological modeling and health surveillance training. However, evidence regarding competency gains remains limited. A recent evaluation by Sutoi et al. (11) demonstrated improvements in self-reported confidence and teamwork skills following interdisciplinary training, but long-term competency outcomes were not assessed.

Recent reforms in Chinese preventive medicine education include competency-oriented programs piloted in Beijing and Shanghai, where students engage in multi-week rotations at local CDCs combined with simulation exercises in infectious disease outbreak response. These initiatives aim to bridge the gap between classroom instruction and practical field experience. In Europe, some public health schools have implemented interdisciplinary modules integrating data science, behavioral science, and health policy, allowing students to work on real-world public health projects. For example, the University of Maastricht's public health program incorporates a multi-country outbreak simulation exercise, giving students exposure to cross-national emergency response.

## 5 The role of practice-based and field-oriented training

Across recent reform efforts, there has been a growing emphasis on strengthening practice-based components within preventive medicine education. This shift reflects a broader recognition that professional competence in public health cannot be developed through theoretical instruction alone (12). Field-based training—such as internships in CDCs, community health institutions, and public health laboratories—is increasingly regarded as essential. For example, students in Beijing and Shanghai have participated in multi-week rotations at provincial and municipal CDCs, where they engage in epidemiological surveillance, outbreak investigation, and vaccination program management. Similarly, community-based placements in local health centers allow students to apply preventive medicine principles in population health monitoring and health promotion activities (13). Internationally, structured field practicums are also widely implemented. For instance, public health students at the Johns Hopkins Bloomberg School of Public Health in the United States complete applied practice experiences within local or state health departments, contributing to epidemiological investigations and public health program evaluations. These examples illustrate both the shared global recognition of practice-based training and the contextual variations in implementation. These experiences demonstrate that immersive, field-based training is critical for developing practical competencies, enhancing decision-making skills, and preparing graduates to respond effectively to real-world public health challenges.

Simulation-based approaches to public health emergencies have also received increasing attention in recent years (14). By exposing students to evolving outbreak scenarios, these training formats can provide opportunities to engage with decision-making processes under conditions that approximate real-world uncertainty. In addition to supporting technical preparedness, such simulations may facilitate the development of interdisciplinary communication and coordination skills. However, their effectiveness is likely to depend on factors such as scenario design, institutional resources, and the extent of faculty involvement. The integration of research training into undergraduate education is gaining prominence as a means of strengthening analytical capacity. Early engagement with research methods may support students in developing skills related to evidence interpretation and critical appraisal, which are increasingly important in data-intensive public health environments (15). The optimal balance between research-oriented training and practice-based skill development remains uncertain. Existing studies have primarily evaluated short-term educational outcomes, while evidence regarding long-term workforce performance remains limited.

Practice-based training models also differ substantially across settings. Chinese institutions increasingly rely on collaborations with CDC systems and community health organizations, whereas many Western programs integrate extended public health practicums, service-learning activities, and multidisciplinary placements throughout training. These approaches reflect different institutional structures but share the objective of strengthening the connection between classroom learning and public health practice.

## 6 Future directions for preventive medicine education

Looking ahead, the future development of preventive medicine education is likely to depend on how effectively training models can respond to increasingly complex and uncertain public health environments. Rather than relying on incremental adjustments, there is a growing need to consider more integrated and adaptive approaches to workforce development.

One area of particular importance is the alignment between educational content and real-world professional demands (16). In many settings, gaps persist between what is taught and what is required in practice. Addressing this mismatch involves not only strengthening technical competencies, but also placing greater emphasis on leadership, communication, and decision-making skills. At the same time, the importance of systems-oriented thinking has become increasingly apparent. Public health challenges rarely operate in isolation; instead, they emerge from interactions between health systems, social determinants, and policy environments. Incorporating these perspectives into training may help students develop a more comprehensive understanding of how interventions function in practice, although doing so in a structured and assessable manner remains challenging.

Another dimension that is receiving growing attention relates to adaptive learning capacity. In the context of rapidly evolving global health threats, the ability to continuously update knowledge and engage with new tools is becoming a core professional requirement. These developments highlight the importance of experiential learning in preparing future public health professionals. However, beyond specific training approaches, broader questions remain regarding how educational systems can cultivate integrated competencies capable of responding to increasingly complex public health environments.

The successful implementation of these educational innovations also depends on faculty development. Beyond disciplinary expertise, educators increasingly require competencies in interdisciplinary teaching, competency-based curriculum design, practice-oriented instruction, and the effective use of digital technologies, including AI–supported educational tools. Faculty members may also need skills in facilitating experiential learning, mentoring students in complex real-world settings, and evaluating higher-order competencies such as systems thinking and adaptive problem-solving.

Given existing resource constraints in many institutions, faculty development initiatives should be designed to be feasible and scalable. Potential approaches include short-term professional development programs, interdisciplinary teaching communities, collaborative partnerships with public health agencies, and targeted training in digital and AI-enhanced pedagogy. Strengthening institutional support for faculty development may be an important prerequisite for the long-term sustainability of preventive medicine education reform. To clarify the relationships among the competencies discussed throughout this review, we propose an integrated competency framework that synthesizes foundational disciplinary knowledge, practice-based skills, interdisciplinary capabilities, adaptive capacities, and emerging digital competencies (Table 1).

**Table 1:** Proposed integrated competency framework for preventive medicine education.

Domain	Core competencies	Educational approaches	Expected outcomes
Foundational public health knowledge	Epidemiology, biostatistics, environmental health	Coursework, case-based learning	Scientific literacy
Practice-based competencies	Field investigation, surveillance, outbreak response	CDC placements, community internships	Workforce readiness
Interdisciplinary competencies	Systems thinking, collaboration, communication	Team-based projects	Cross-sector problem solving
Adaptive and post-competency capacities	Adaptability, reflective learning, uncertainty management	Scenario simulations	Resilience and flexibility
Digital and AI competencies	Data analytics, AI literacy, digital health	AI-supported learning activities	Technology-enabled practice

Among these domains, digital and AI-related competencies represent a particularly dynamic area of development and warrant further discussion.

## 7 Emerging role of AI in preventive medicine education

### 7.1 Curriculum design

Recent developments in AI are beginning to influence multiple aspects of public health practice, with corresponding implications for preventive medicine education (17). While earlier applications have largely focused on predictive modeling and surveillance, more recent work has explored systems capable of iterative reasoning and task-oriented execution, sometimes referred to as AI (18). These systems are designed to operate in data-rich and dynamic environments, where they can support processes such as planning, adaptation, and decision-making. Empirical evidence remains relatively limited. Acosta highlighted the growing importance of AI literacy in public health education but noted the lack of rigorous evaluations linking AI-based educational interventions to measurable workforce competencies (19).

Within educational settings, AI-based tools have been explored as a means of supporting training in areas such as epidemiological analysis, data interpretation, and decision-making. For example, machine learning models can be used to construct simulated outbreak scenarios, providing learners with opportunities to engage in real-time analysis of transmission patterns and intervention strategies. Similarly, natural language processing (NLP) techniques have been applied to extract structured information from unstructured health data, which may help students navigate large and complex datasets (20).

Agentic AI may further extend existing digital approaches by enabling more interactive and adaptive learning environments. Compared with conventional tools, these systems have the potential to function not only as information providers but also as active participants in problem-solving processes. In educational settings, this could allow students to engage with complex public health scenarios in a more iterative manner—for example, by formulating and revising hypotheses or exploring alternative intervention strategies (21). Such features appear broadly consistent with the objectives of competency-oriented training, particularly in relation to skills such as systems thinking and decision-making under conditions of uncertainty. However, how consistently these capabilities translate into meaningful learning outcomes remains to be determined. The increasing interest in AI within preventive medicine education is also closely linked to the broader shift toward data-driven and precision-oriented public health approaches (22). As analytical tools become more embedded in routine practice, there is a growing expectation that future professionals will be able to engage with large-scale data and computational methods. In this context, exposure to AI-related tools during training may provide a useful foundation. At the same time, it is not yet clear to what extent such exposure leads to sustained competency development, particularly in settings where technological infrastructure and instructional support vary.

### 7.2 Assessment and competency development

Post competency has been described as the capacity to adapt knowledge, skills, and professional judgment in complex, uncertain, and rapidly changing environments (23). Importantly, the integration of AI and agentic systems also introduces a range of challenges that extend beyond technical implementation. Concerns related to data quality, algorithmic bias, interpretability, and ethical governance have been widely discussed, and their implications for education are only beginning to be explored (24). There is a risk that an overemphasis on automated tools could inadvertently weaken critical thinking if not carefully managed. For this reason, the role of AI in preventive medicine training may be better understood as complementary rather than substitutive. Incorporating elements such as AI literacy, ethical reflection, and human–AI collaboration into curricula is likely to be important, although optimal approaches to doing so remain under discussion (25). These developments suggest that AI—particularly in its more agentic forms—has the potential to reshape aspects of preventive medicine education. At the same time, its long-term value will depend on how it is integrated into training systems and whether it can support, rather than displace, the development of core public health competencies (26).

## 8 Limitations

Several limitations should be acknowledged. First, as a mini review, this article does not provide a fully systematic synthesis of all available literature, and study selection was guided by relevance to the review objectives. Second, while efforts were made to incorporate both Chinese and international perspectives, the availability and comparability of evidence varied across regions and topics. Third, some of the emerging themes discussed, particularly those related to AI in preventive medicine education, are based on a rapidly evolving evidence base for which long-term educational outcomes remain incompletely understood. These limitations should be considered when interpreting the conclusions and recommendations presented in this review.

## 9 Conclusion

Preventive medicine education is undergoing significant transformation in response to evolving public health challenges following the COVID-19 pandemic. Although national contexts differ in policy priorities, educational structures, and workforce needs, many reform efforts demonstrate a growing convergence toward competency-based, practice-oriented, and technology-enabled training models. While substantial progress has been made in competency-oriented training, interdisciplinary integration, and technology-enabled learning, important challenges remain in aligning educational models with workforce needs and public health practice.

Future reforms will likely depend on strengthening systems-oriented thinking, expanding meaningful practice-based learning opportunities, and critically integrating emerging technologies such as AI. Continued evaluation of educational innovations across different contexts will be essential to ensure that preventive medicine training supports a resilient and adaptable public health workforce.

Ultimately, the extent to which preventive medicine education can adapt to evolving public health needs remains uncertain. Continued evaluation and context-specific adaptation are critical to preparing graduates for the evolving challenges of public health.

## References

[1] Connell L Finn Y Sixsmith J. Health literacy education programmes developed for qualified health professionals: a scoping review. BMJ Open. (2023) 13:e070734. doi: 10.1136/bmjopen-2022-070734 36997248 PMC10069593

[2] Li H Chen C Chen A Lin Q Li D Chen M . Effect of a three-years preventive medicine vocational education program on county-level healthcare workforce development in China: a cross-sectional study. BMC Med Educ. (2025) 25:522. doi: 10.1186/s12909-025-07095-w 40217268 PMC11992889

[3] Gichane MW Wallace DD. Dismantling and reimagining global health education. Glob Health Action. (2022) 15:2131967. doi: 10.1080/16549716.2022.2131967 36285634 PMC9621231

[4] Lee GB Chiu AM. Assessment and feedback methods in competency-based medical education. Ann Allergy Asthma Immunol. (2022) 128:256–62. doi: 10.1016/j.anai.2021.12.010 34929390

[5] Schwitz F Brodmann Maeder M Hennel EK. Competency-based education - the reform of postgraduate medical training in Switzerland. GMS J Med Educ. (2024) 41:Doc62. doi: 10.3205/zma001717 39711866 PMC11656186

[6] Lyu S Qian C Yuan L Yuan Z Lee C-H. Health system resilience and pandemic response: a comparative analysis of China, Singapore, the U. S., and the UK. Front Public Health. (2025) 13:1666323. doi: 10.3389/fpubh.2025.1666323 40959645 PMC12434018

[7] Magaña-Valladares L Penniecook T. The future of public health education: the post covid-19 era. Salud Publica Mex. (2022) 64:551–5. doi: 10.21149/13949 36750075

[8] Davies AJ Shepherd I Leigh E. Enhancing leadership training in health services - an evidence-based practice-oriented approach. Leadersh Health Serv. (2022) ahead-of-print. doi: 10.1108/LHS-04-2022-0040 35766369

[9] Tarfa A Fadul N Stohs E Wetherhold J Kebede M Mirghani N . Providing education and training to health care professionals to address COVID-19 health disparities: protocol for implementation project using reach, effectiveness, adoption, implementation, and maintenance framework. JMIR Res Protoc. (2025) 14:e60901. doi: 10.2196/60901 40377970 PMC12125559

[10] Oliver J Ferdinand A Hussein A Hussein R Kaufman J Edler P . Evaluating a peer-to-peer health education program in Australian public housing communities during the COVID-19 pandemic. BMC Health Serv Res. (2024) 24:250. doi: 10.1186/s12913-024-10627-7 38413968 PMC10900559

[11] Sutoi D Popa DI Cindrea CA Trebuian CI Williams C Sutoi M . The impact of a one-day multidisciplinary workshop on medical students' self-assessed confidence, knowledge, and teamwork skills: a pre-post study. Adv Med Educ Pract. (2025) 16:401–10. doi: 10.2147/AMEP.S509297 40166603 PMC11955773

[12] Cao YL Shan J Bao XY Jin M Huai W Jin YC . Global landscape and professionalization of infection preventionist education: comparative insights and future directions. Front Public Health. (2025) 13:1736787. doi: 10.3389/fpubh.2025.1736787 41584190 PMC12827665

[13] Easterling D Perry AC Woodside R Patel T Gesell SB. Clarifying the concept of a learning health system for healthcare delivery organizations: implications from a qualitative analysis of the scientific literature. Learn Health Syst. (2022) 6:e10287. doi: 10.1002/lrh2.10287 35434353 PMC9006535

[14] Fang X Zhao L Pang R Li H Ye P. Responsibility of education in improving medical college students' ability to prevent and respond to public health emergencies in China - a systematic review. Front Public Health. (2023) 11:1191723. doi: 10.3389/fpubh.2023.1191723 38125842 PMC10731453

[15] McFadden BR Reynolds M Inglis TJJ. Developing machine learning systems worthy of trust for infection science: a requirement for future implementation into clinical practice. Front Digital Health. (2023) 5:1260602. doi: 10.3389/fdgth.2023.1260602 37829595 PMC10565494

[16] Gorga A Barbati C Airapetian M Briganti GL Carnevali ED Cervellera F . Mapping digital public health training: are we preparing the European workforce? Front Public Health. (2026) 14:1778953. doi: 10.3389/fpubh.2026.1778953 41756088 PMC12932469

[17] Dyar OJ Haglund BJA Melder C Skillington T Kristenson M Sarkadi A. Rainbows over the world's public health: determinants of health models in the past, present, and future. Scand J Public Health. (2022) 50:1047–58. doi: 10.1177/14034948221113147 36076363 PMC9578089

[18] Mahl D Schäfer MS Voinea SA Adib K Duncan B Salvi C . Responsible artificial intelligence in public health: a Delphi study on risk communication, community engagement and infodemic management. BMJ Glob Health. (2025) 10:e018545. doi: 10.1136/bmjgh-2024-018545 40409762 PMC12104889

[19] Acosta JA. Perspective: advancing public health education by embedding AI literacy. Front Digital Health. (2025) 7:1584883. doi: 10.3389/fdgth.2025.1584883 40741324 PMC12307283

[20] Hu S Oppong A Mogo E Collins C Occhini G Barford A . Natural language processing technologies for public health in Africa: scoping review. J Med Internet Res. (2025) 27:e68720. doi: 10.2196/68720 40053738 PMC11923465

[21] Lee Y-CJ Takenaka BP. Extended reality as a means to enhance public health education. Front Public Health. (2022) 10:1040018. doi: 10.3389/fpubh.2022.1040018 36504953 PMC9726919

[22] Aggarwal A Tam CC Wu D Li X Qiao S. Artificial intelligence-based chatbots for promoting health behavioral changes: systematic review. J Med Internet Res. (2023) 25:e40789. doi: 10.2196/40789 36826990 PMC10007007

[23] Saber M Ebrahimi S Farzane N Shakeri A. Use of simulated patients for formative assessment of moral competence in medical students. J Educ Health Promot. (2022) 11:330. doi: 10.4103/jehp.jehp_1275_21 36567988 PMC9768714

[24] Bose S Prakash A Prusty AK Verma R Padmavathy K Iragamreddy VR. Artificial intelligence (AI) supported decision-making in intensive care units: implications for nursing and medical practice. Cureus. (2026) 18:e104266. doi: 10.7759/cureus.104266 41909315 PMC13031828

[25] Gundlack J Negash S Thiel C Buch C Schildmann J Unverzagt S . Artificial intelligence in medical care - patients' perceptions on caregiving relationships and ethics: a qualitative study. Health Expect. (2025) 28:e70216. doi: 10.1111/hex.70216 40094179 PMC11911933

[26] Mair JL Hashim J Thai L Tai ES Ryan JC Kowatsch T . Understanding and overcoming barriers to digital health adoption: a patient and public involvement study. Transl Behav Med. (2025) 15:ibaf010. doi: 10.1093/tbm/ibaf010 40167046 PMC11959363

